# Experimental study of the vascular normalization window for tumors treated with apatinib and the efficacy of sequential chemotherapy with apatinib in lung cancer‐bearing mice and patients

**DOI:** 10.1002/cam4.2923

**Published:** 2020-02-19

**Authors:** Mingtao Liu, Hui Li, Xiuxiu Wang, Lijun Jing, Peng Jiang, Yu Li

**Affiliations:** ^1^ Department of Pulmonary Medicine Qilu Hospital Shandong University Jinan Shandong China; ^2^ Department of Pulmonary Medicine Binzhou People^’^s Hospital Binzhou Shandong China; ^3^ Department of Pulmonary Medicine Weihai Municipal Hospital Weihai China

**Keywords:** Apatinib, chemotherapy, Combined therapy; vascular normalization

## Abstract

In the tumor vascular system, the vascular structure is disordered, the morphology is abnormal, and the structure of the blood vessel walls is incomplete, leading to leakage of the blood vessel wall, elevated interstitial fluid pressure, and elevated blood flow resistance. These alterations lead to local microenvironmental changes, which mainly manifest as a lack of oxygen and acidosis, further affecting the efficacy of chemotherapy drugs. Antiangiogenic drugs can normalize the abnormalities caused by tumor angiogenesis, thereby transferring oxygen and drugs to tumor cells more efficiently through normalized blood vessels and enhancing the efficacy of chemotherapy drugs. Apatinib is a specific VEGFR‐2 inhibitor that blocks the transmission of the VEGF/VEGFR‐2 signaling pathway. In this study, we constructed a nude mouse xenograft model of lung cancer and administered apatinib at different doses and times to detect the normalization of reactive blood vessels through VEGF, α‐SMA, college‐IV, HIF‐1α, and MMP. The ultrastructure of tumor blood vessels was observed by electron microscopy, and the dose and timing of apatinib‐induced normalization of lung cancer in nude mice were confirmed. Then, we observed the inhibitory effect of apatinib combined with pemetrexed on transplanted tumors of lung cancer cells in nude mice at different time points and observed whether combination pemetrexed chemotherapy showed more significant effects in the time window of vascular normalization induced by apatinib. The inhibition of the growth of transplanted tumors was examined. Then 20 patients with advanced non–small cell lung cancer were enrolled, and apatinib sequential chemotherapy drugs were applied as a third‐line chemotherapy regimen to observe its clinical efficacy.

## Graphical abstract

In this work, we confirmed that low doses of apatinib can induce tumor vascular normalization within a certain period of time, and combined chemotherapy can improve the therapeutic effect during this period of time.

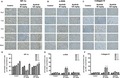

## 1 INTRODUCTION

Lung cancer is the leading cause of cancer‐related death worldwide, with approximately 1.2 million deaths annually.^1^ More than 80% of lung cancer cases are of non–small cell cancer (NSCLC).^2^ Approximately 51% of patients present with advanced disease at diagnosis.^3^ The standard of care in patients with advanced disease is platinum‐based doublet chemotherapy.^4^ Although chemotherapy has improved the outcome and quality of life of patients, the prognosis remains unfavorable, with a median survival time that does not exceed 10 months.^5^, ^6^, ^7^ Improving the efficacy of treatments for patients with lung cancer and prolonging their survival is an urgent issue for clinicians.

Folkman proposed that tumor growth depends on the formation of tumor blood vessels and thus proposed that antiangiogenic strategies will be important for the treatment of tumors.^8^ The traditional view was that antiangiogenic drugs can reduce the formation of tumor blood vessels, cause tumor necrosis, and "starve" tumors.^9^ However, in recent years, many preclinical and clinical studies have shown that the short‐term application of antiangiogenic drugs can have therapeutic effects, whereas long‐term use can lead to vascular necrosis in the central area of the tumor, which results in hypoxia and an acidic microenvironment and decreases the sensitivity of solid tumor cells to chemotherapy.^10^, ^11^ In response to these clinical problems, Jain proposed the tumor vascular normalization theory, which suggests that tumor angiogenesis is a complex process in which the imbalance between proangiogenic factors and angiogenesis inhibitors is a key factor.^12^, ^13^ In tumor tissues, a variety of transcription factors, such as hypoxia‐inducible factor (HIF), can trigger an increase in proangiogenic factors.^14^ The most important proangiogenic factor is vascular endothelial growth factor (VEGF), which leads to an increase in neovascularization.^15^ The neovascular morphology is distorted, swollen, and cystic. The morphology of pericytes is abnormal, their function is insufficient, the connections are loose or even absent, and the basement membrane is incomplete and uneven.^16^ The abnormal structure and function of tumor blood vessels lead to local blood flow disorders, and loss of the vascular wall barrier function leads to local blood leakage.^17^ These changes result in an increase in interstitial pressure (IFP) and further impede the blood flow perfusion of tumor tissues, resulting in tumor cells with hypoxia, a low pH, and a tumor microenvironment with a high IFP, which interferes with the diffusion of chemotherapeutic drugs in tumor cells and reduces the efficacy of chemotherapy drugs.^18^ Reasonable applications of antiangiogenic drugs can normalize the abnormally distorted tumor blood vessels and the vascular structure, result in an intact vascular basement membrane, increase the supporting cells around the blood vessels, enhance the vascular supply of nutrients, and promote efficient transport through the blood vessels.^19^ The delivery of oxygen and drugs to tumor cells improves the efficacy of chemotherapy.

At present, research on the normalization of antiangiogenic drugs has mainly focused on bevacizumab.^20^, ^21^ Dickson used bevacizumab, an inhibitor of angiogenesis that neutralizes human VEGF, to examine vascular normalization in mice bearing human neuroblastoma xenografts. The results revealed that the vessels showed normalization within 24 hours of therapy, including reductions in microvessel density (MVD) and vessel length, diameter, and tortuosity, vascular permeability, and tumor IFP and an improvement in perfusion.^22^ Moreover, this treatment improved the delivery of chemotherapeutics to the tumors. Moreover, clinical studies of bevacizumab combined with chemotherapy drugs for advanced lung cancer have also confirmed that chemotherapy drugs combined with bevacizumab can effectively improve the progression‐free survival (PFS) and overall survival (OS) of patients with advanced lung cancer.^23^, ^24^

Apatinib is a small molecule tyrosine kinase inhibitor that selectively inhibits the phosphorylation between VEGFR‐2 and tyrosine by competitively binding to the VEGFR‐2 intracellular tyrosine ATP binding site. Kinase activity blocks the transmission of the VEGF/VEGFR‐2 signaling pathway and inhibits tumor angiogenesis, thereby inhibiting tumor growth.^25^, ^26^ At present, apatinib is mainly used in the treatment of advanced gastric cancer as a third‐line or higher treatment.^27^ Furthermore, a series of clinical trials of apatinib in the treatment of advanced lung cancer initially confirmed that apatinib can effectively slow the growth of lung cancer. The clinical results of apatinib confirmed that patients can benefit in the short term, but resistance developed rapidly, and improved long‐term OS was not obvious. The effect of apatinib combined with chemotherapy on the OS of patients was not obvious.^28^, ^29^ An important reason may be the timing of the combination chemotherapy drugs. We expected apatinib to induce tumor vascular normalization, and combined chemotherapy was assessed in the vascular normalization window to improve the efficacy of chemotherapy drugs. Therefore, a study on the vascular normalization of apatinib is especially important. In this study, a nude mouse xenograft model was used to determine the optimal dose and duration of apatinib‐induced tumor vascular normalization by detecting a series of indicators of normalization of vascularization.^19^ Then, we examined whether combination chemotherapy in the normalization time window of blood vessels can effectively improve the therapeutic effect.

## 2 MATERIALS AND METHODS

### 2.1 Cell culture

The human lung cancer cell line A549 were purchased from the Cell Bank of the Chinese Academy of Sciences (Shanghai, China). A549 cells were maintained in RPMI‐1640 (HyClone) supplemented with 10% FBS (Gibco, NY), 100 U/mL penicillin (HyClone), 50 mg/mL streptomycin (HyClone), and 2 mmol/L glutamine in a humidified CO_2_ incubator at 37°C. Cells were passaged for less than 3 months before renewal from frozen, early‐passage stocks obtained from the indicated sources.

### 2.2 Reagents and antibodies

Apatinib mesylate (Heng Rui) were grinded into powder and dissolved 0.5%CMC (Solrbio). Pemetrexed (Qi Lu) were dissolved 0.9% saline. Primary antibodies against AKT (ab8805), phospho‐AKT (ab8932), ERK (ab54230), phospho‐ERK (ab201015), mTOR (ab2732), phospho‐mTOR (ab84400), MEK (ab178876), phospho‐MEK (ab194754), HIF‐1α (ab51608), CD31 (ab28364), α‐SMA (ab5694), collagen IV (ab6586), MMP2 (ab37150), MMP‐9 (ab38898), and β‐actin (ab8227) were purchased from abcam. Primary antibodies against cleaved‐caspase3 (9664), ki67 (9449), and anti‐rabbit or anti‐mouse IgG horseradish peroxidase (HRP)‐linked secondary antibodies were purchased from Cell Signaling Technology.

### 2.3 Tumor xenograft mouse models

Four‐ to 5‐week‐old female BALB/c nude mice were purchased from Nanjing University Biomedical Research Institute (Nanjing, China). All animal experiments were performed in the animal research center of Shandong Medical Academy in accordance with the National Institutes of Health guide for the care and use of laboratory animals. A549 cells were selected to construct the nude mouse xenograft model. Approximately 2 × 10^6^ cells were suspended in 0.2 mL of PBS and subcutaneously injected into the right chest wall of each nude mouse, and tumors formed in approximately 10 days. The tumor volume was calculated as L × W^2^/2 (L and W are the length and width of the tumor, respectively). The nude mice with transplanted tumor volumes of 50–150 mm^3^ were selected for intervention grouping. There was no significant difference in the average volume of transplanted tumors between the nude mice before intervention.

In the first step, 60 tumor‐bearing nude mice for vascular normalization studies were randomly divided into three groups according to a random number table. The animals were treated for 10 consecutive days once daily by oral gavage with normal saline and apatinib (60 and 120 mg/kg). The dose of apatinib refers to previously published literature^30^ and our pre‐experimental study.^31^ The 60 and 120 mg/kg were defined as low‐ and high‐dose groups, respectively. (Specific references were included here). Four nude mice were sacrificed in each group before administration and on the first day, the third day, the seventh day, and the tenth day after administration.

In the second step, 32 tumor‐bearing nude mice were randomly divided into four groups according to a random number table, and eight nude mice were in each group. (1) Control group: injected with a volume of normal saline using the same method. (2) Pemetrexed group: abdominal cavity injection of pemetrexed at 150 mg/kg. (3) Apatinib and pemetrexed synchronization group: oral gavage with apatinib at a daily dose of 60 mg/kg for 14 days, followed by abdominal cavity injection of pemetrexed on the first day and the dose as before. (4) Apatinib sequential pemetrexed group: Apatinib was given for a certain period of time to induce normalization of the blood vessels, and then, the abdominal cavity administration of pemetrexed was given during the window of normalization of the blood vessels, with the same dose as before, and the time to induce normalization of blood vessels was determined according to the results of the first step.

The nude mice were treated for 14 consecutive days. The body weight and the tumor volume were recorded every 3 days by the same person. At harvest, the mice were sacrificed under anesthesia and were photographed. The tumor tissues were fixed in 4% paraformaldehyde for immunohistochemistry or stored at −80°C for Western blotting. The time and dose of apatinib‐induced vascular normalization were determined by detecting a series of indicators in nude mouse xenografts by immunohistochemistry, Western blots, and electron microscopy. All animal experiments were performed with the approval of the Shandong University Animal Care and Use Committee. The schematic diagram of the experimental design of BALB/c nude mice was shown in figure 4A for details.

### 2.4 Western blot analysis

The transplanted tumor tissues were lysed by radio immunoprecipitation assay (RIPA, Beyotime, China) buffer containing phenyl methane sulfonyl fluoride (PMSF, Solarbio, China) with mild sonication. The concentrations of total proteins were measured by the BCA Protein Assay Kit (Beyotime, China). Equal amount of protein was subjected to 10% sodium dodecyl sulfate polyacrylamide gel electrophoresis (SDS‐PAGE) and blotted on polyvinylidene fluoride (PVDF, Millipore, Billerica, USA). Protein bands were visualized via enhanced chemiluminescence (ECL, Millipore, USA) and were analyzed using the western blot imaging system (AI600 images, GE, USA), followed by measurement of the density of each band using Image J software.

### 2.5 Immunohistochemical staining

For immunohistochemical staining, the transplanted tumor tissues were fixed in 4% paraformaldehyde, and paraffin blocks were prepared after dehydrating and embedding. A series of 3 mm sections were obtained from each paraffin block. Each slice was baked at 65°C for 1 hours and dewaxed by xylene. Then, the slides were dehydrated using ethanol and antigens were repaired by EDTA. The peroxidase was removed using 3% H_2_O_2_ and blocked using 5% BSA for 30 min. Incubation with the primary antibody was conducted overnight at 4°C. The sections were incubated with a biotin secondary antibody for 20 minutes at room temperature. Targeted proteins were visualized using the peroxidase substrate diaminobenzidine. Staining intensities were estimated in five random fields per section by three independent observers individually.

### 2.6 Electron microscope specimen production

The transplanted tumor tissues of the mice were fixed in 3% glutaraldehyde at 4°C for 4 hours and then washed with 0.1‐M sodium dicarboxylate buffer and soaked in 1% citric acid at 4°C for 2 hours. Next, the tissues were washed with 0.1‐M sodium diformate buffer twice and were dehydrated by graded ethanol. The tissues were permeated with propylene oxide, completely embedded in an embedding solution, and placed in a 40°C incubator for 12 hours. Then, the slides were transferred to an embedding plate and incubated at 60°C for 48 hours. After the fabrication, the ultrastructure of the organ was observed by transmission electron microscopy (JEOL).

### 2.7 Patients, treatment, and evaluation

Twenty eligible patients who were diagnosed with advanced non–small cell lung cancer by pathological biopsy, all of whom had wild‐type EGFR, from January 2017 to October 2018 in our hospital. Patients had first‐ and second‐line chemotherapy failure, lesion progression, and application of apatinib sequential chemotherapy drugs, such as docetaxel, as a third‐line chemotherapy regime. Apatinib (250 mg/day) was administered at a lower than normal oral dose for 5 days, followed by pemetrexed or docetaxel chemotherapy, and every 28 days was one cycle. This study was conducted after approval of the hospital's Ethics Committee (Ethical code: 2 018 166).

### 2.8 Statistical analysis

All data are expressed as the mean ± standard error of the mean (SEM). Data were analyzed using GraphPad Prism software, version 5.0. A two‐tailed unpaired Student's t‐test was performed to compare two datasets. Multiple comparisons were tested with two‐way ANOVA followed by Bonferroni's post‐test. A P‐value of less than 0.05 was considered statistically significant.

## 3 RESULTS

### 3.1 Detection of vascular normalization‐related indicators

**Figure 1:**
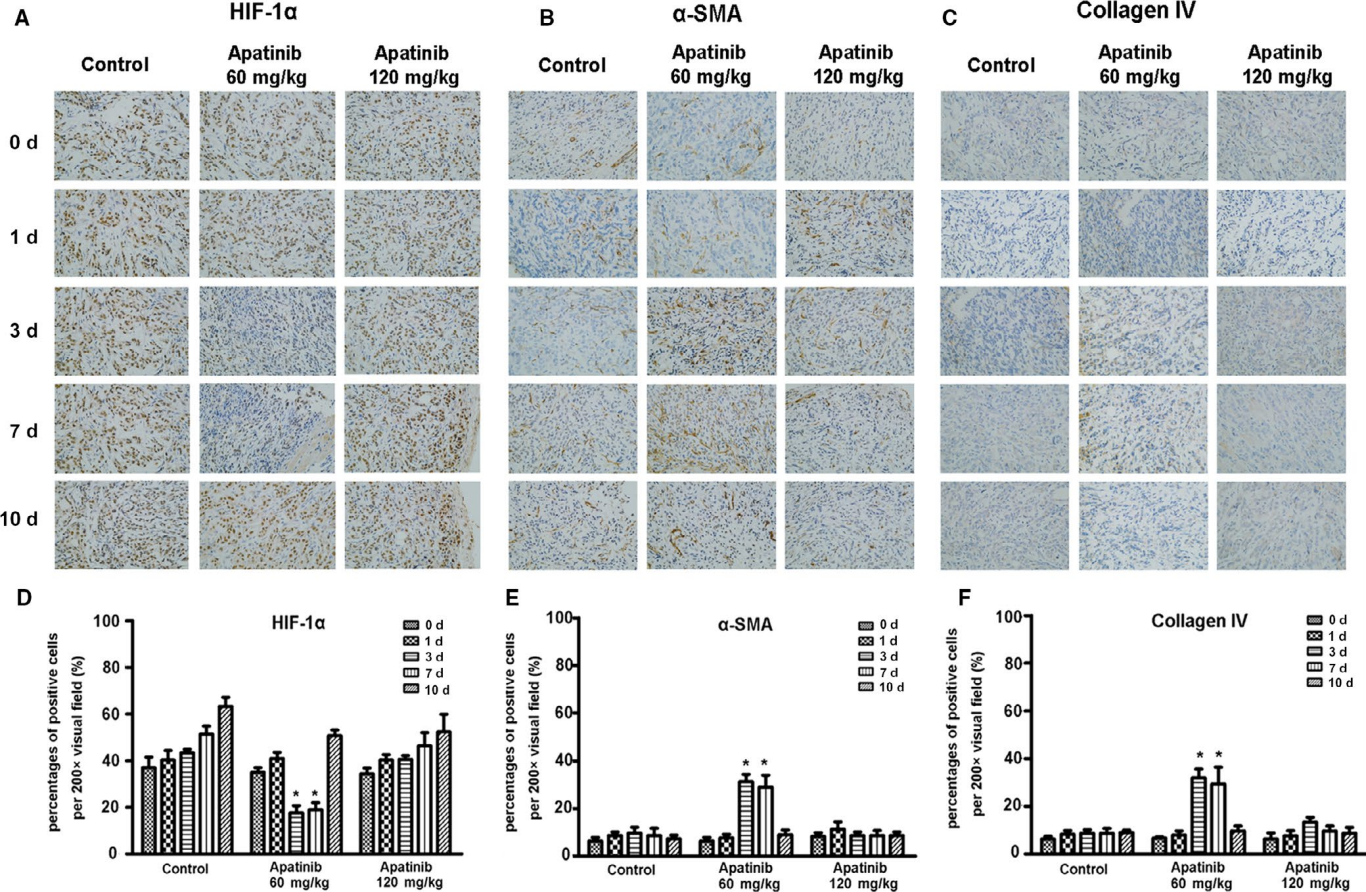
The immunohistochemical staining of the expression of HIF‐1α, α‐SMA, and collagen IV in different groups of xenografted‐tumor tissues after administration of apatinib at different concentration and time. (A, B, and C) The expression of HIF‐1α, α‐SMA, and collagen IV was detected by immunohistochemical staining. (D, E, and F) The quantifications of HIF‐1α, α‐SMA, and collagen IV in each group

In tumor tissues, hypoxia‐inducible factors can induce an increase in proangiogenic factors, leading to an increase in neovascularization, variability in neovascular morphology, sac‐like morphological changes in pericytes, insufficient function, an incomplete basement membrane, and uneven thickness. Antiangiogenic drugs can normalize tumor blood vessels and, thus, the first improvement is the hypoxic state of the tumor tissues. Therefore, the expression of hypoxia‐inducible factors can be indicators of tumor vascular normalization. Moreover, α‐SMA and type IV collagen indicate the condition of the pericytes and vascular basement membrane. The expression level reflects the maturity of blood vessels. The higher the expression level, the more mature the blood vessels and the more complete the structure is. The results showed that after treatment with apatinib in the low‐dose group, HIF‐1α was significantly decreased on the 3rd and 7th days of treatment (Figure 1A), whereas the α‐SMA and type IV collagen levels were significantly increased compared with those at other times; there was a statistically significant difference between the groups (*P* < .05) (Figure 1B,C). However, in the high‐dose apatinib group, there were no significant differences in the expression of HIF‐1α, α‐SMA, and IV‐collagen between groups at each time point (*P* > .05) (Figure 1D‐F).

### 3.2 Observation of tissue vascular ultrastructure by electron microscopy

**Figure 2:**
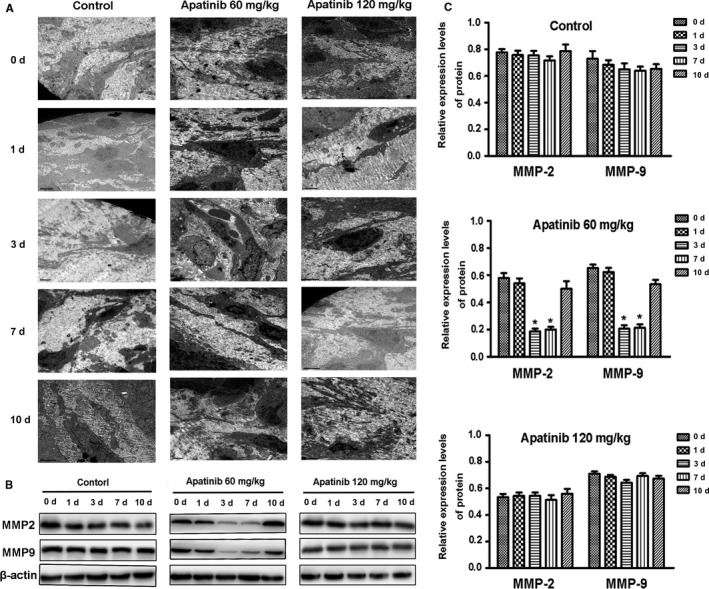
The ultrastructure of xenografted‐tumor vessels under electron microscope and the expression of MMP2 and MMP9 protein in different groups after administration of apatinib at different concentration and time. A, The ultrastructure of xenografted‐tumor vessels under electron microscope. B, The expression of MMP2 and MMP9 protein in different groups was detected by Western blotting. Blots are representative of three independent experiments (C ‐ E) The quantifications of the associated gray protein intensities of MMP2 and MMP9 in each group are presented

Ultrastructural observation of the vascular tissue in transplanted tumors treated with different doses and timing of apatinib was observed under electron microscopy. On the 3rd and 7th days of low‐dose apatinib treatment, the vascular structure in the transplanted tumor tissues was complete and regular in shape, the pericyte cell structure was observed, the vascular basement membrane structure was intact, and no obvious gaps were observed. While the vascular structure was still disordered, no mature pericyte cell structures were observed, and the vascular basement membrane was incomplete at other time points and in the high‐dose groups (Figure 2A).

### 3.3 Expression of MMP‐2 and MMP‐9 in transplanted tumor tissues

Hypoxia is a common event in solid tumors, and HIF‐1α is a major regulator of perception and response to hypoxia. This molecule stimulates the secretion of the proangiogenic factor VEGF, which is a major factor in stimulating tumor angiogenesis. This factor not only promotes the survival of vascular endothelial cells and inhibits apoptosis but also stimulates the secretion of MMP. In particular, MMP‐2 and MMP‐9 were critical for the degradation of basement membrane type IV collagen and increased vascular leakage and interstitial fluid pressure (IFP). In this study, the expression levels of MMP‐2 and MMP‐9 in transplanted tumor tissues were significantly decreased during the 3‐7 days of low‐dose apatinib treatment compared with those in other groups (*P* < .05). There were no significant changes in the expression levels at other times or in the high‐dose group (*P* > .05) (Figure 2B,C).

### 3.4 The microvessel density (MVD) in transplanted tumor tissues

**Figure 3:**
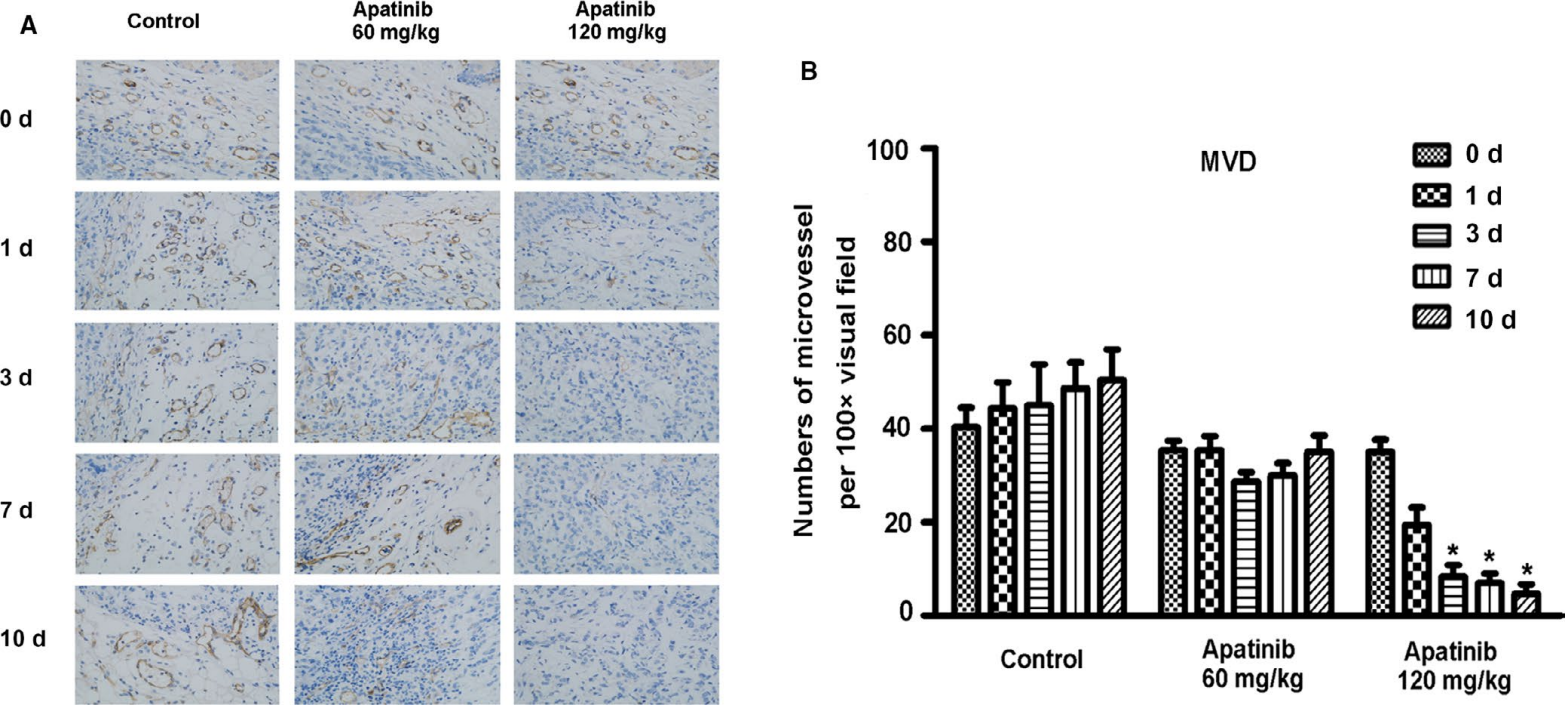
The immunohistochemical staining of the expression of MVD in different groups of xenografted‐tumor tissues after administration of apatinib at different concentration and time (A) and the quantifications of the expression of MVD in each group (B)

Why does high‐dose apatinib treatment fail to induce vascular normalization in transplanted tumors? We hypothesized that high doses of apatinib significantly inhibited the expression of microvessels in transplanted tumor tissues. To further confirm this view, we detected MVD in transplanted tumor tissues with an immunohistochemical analysis. The results showed that the MVD in the control group gradually increased and MVD decreased slightly on the 3rd to 7th day after treatment in the low‐dose apatinib group. There were no statistically significant differences at the other time periods. In the high‐dose apatinib group, the MVD in transplanted tumor tissues was significantly reduced with the application of apatinib (Figure 3A,B). This finding explains why low‐dose apatinib could induce vascular normalization, whereas high‐dose apatinib significantly destroyed the structure of blood vessels in the transplanted tumor tissue and resulted in a significant reduction in blood vessels in the tumor tissue; thus, there was no time window for the normalization of blood vessels.

### 3.5 Inhibitory effect of apatinib combined with chemotherapy on transplanted tumors

**Figure 4:**
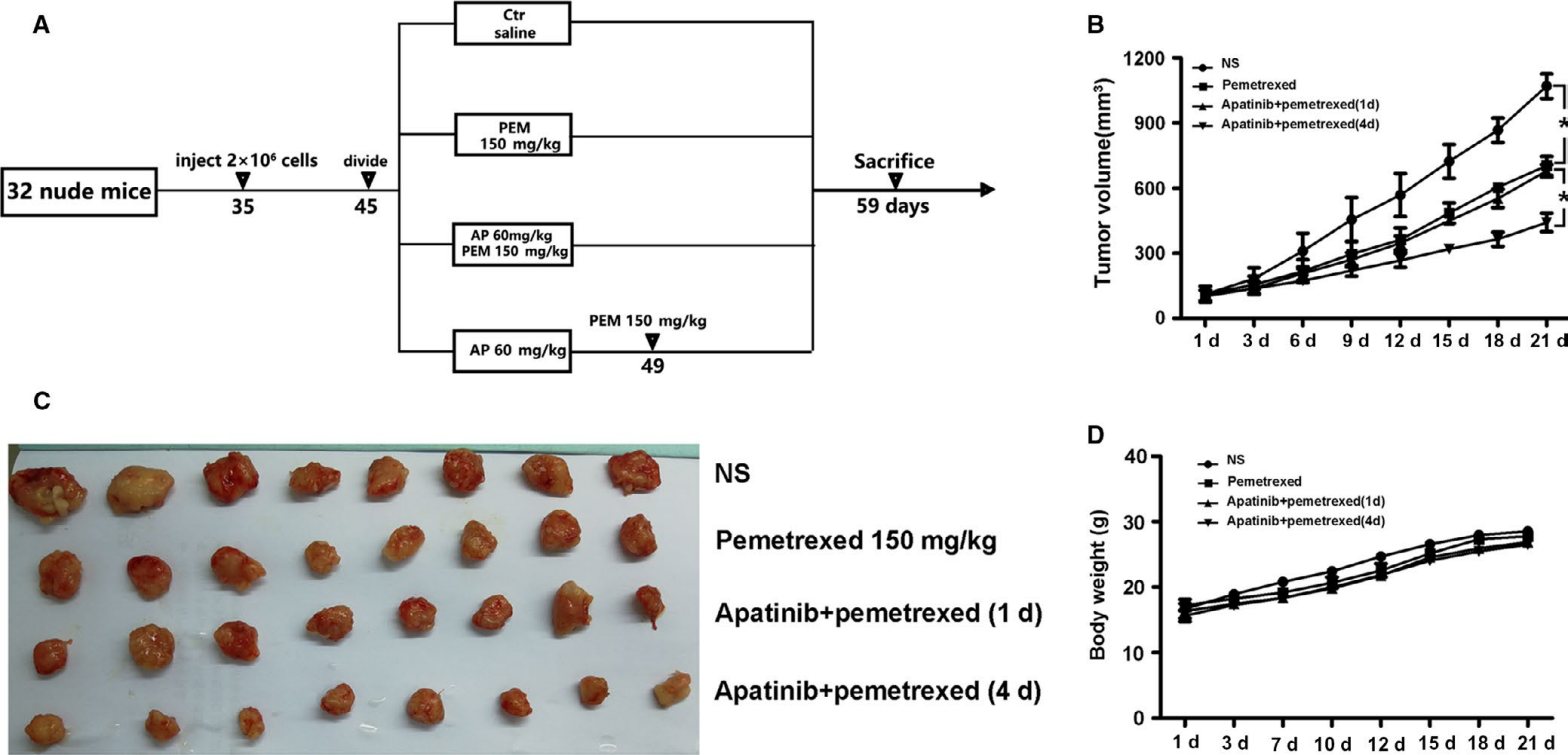
Different modes of administration inhibited tumor growth in A549 xenograft models. (A) Experimental design of experimental protocols in BALB/c nude mice. 35‐day‐old mice were subcutaneously injected with A 549 cells. When the tumor volumes reached 50‐150 mm^3^ at day 45, tumor bearing was treated with vehicle, pemetrexed (150 mg/Kg), apatinib 60 mg/kg combined with pemetrexed 150 mg/Kg at the first day and apatinib 60 mg/kg combined with pemetrexed 150 mg/Kg at the fourth day. 14 days after injection, the mice were sacrificed to determine tumor volumes and were photographed. (B) The tumor growth curves (C). The xenografted‐tumor image. (D).The changes of body weight over 21 days in each group

Based on the therapeutic effect, the inhibitory effect of sequential therapy with low‐dose apatinib and pemetrexed was the best, whereas low‐dose apatinib with synchronous pemetrexed treatment was not superior to pemetrexed monotherapy (Figure 4B). From the tumor inhibition curve, the three groups showed significantly inhibited growth of the transplanted tumors compared with the control group. There was a significant difference in tumor volume, and sequential therapy with low‐dose apatinib and pemetrexed resulted in the best inhibitory effect, which was significantly different from that of synchronous treatment (Figure 4C). There were no significant differences in the body weights of the nude mice in each group, indicating the high safety of all intervention methods. (Figure 4D).

### 3.6 The expression of Ki67, caspase3, and related signaling pathway factors in transplanted tumor tissues

**Figure 5:**
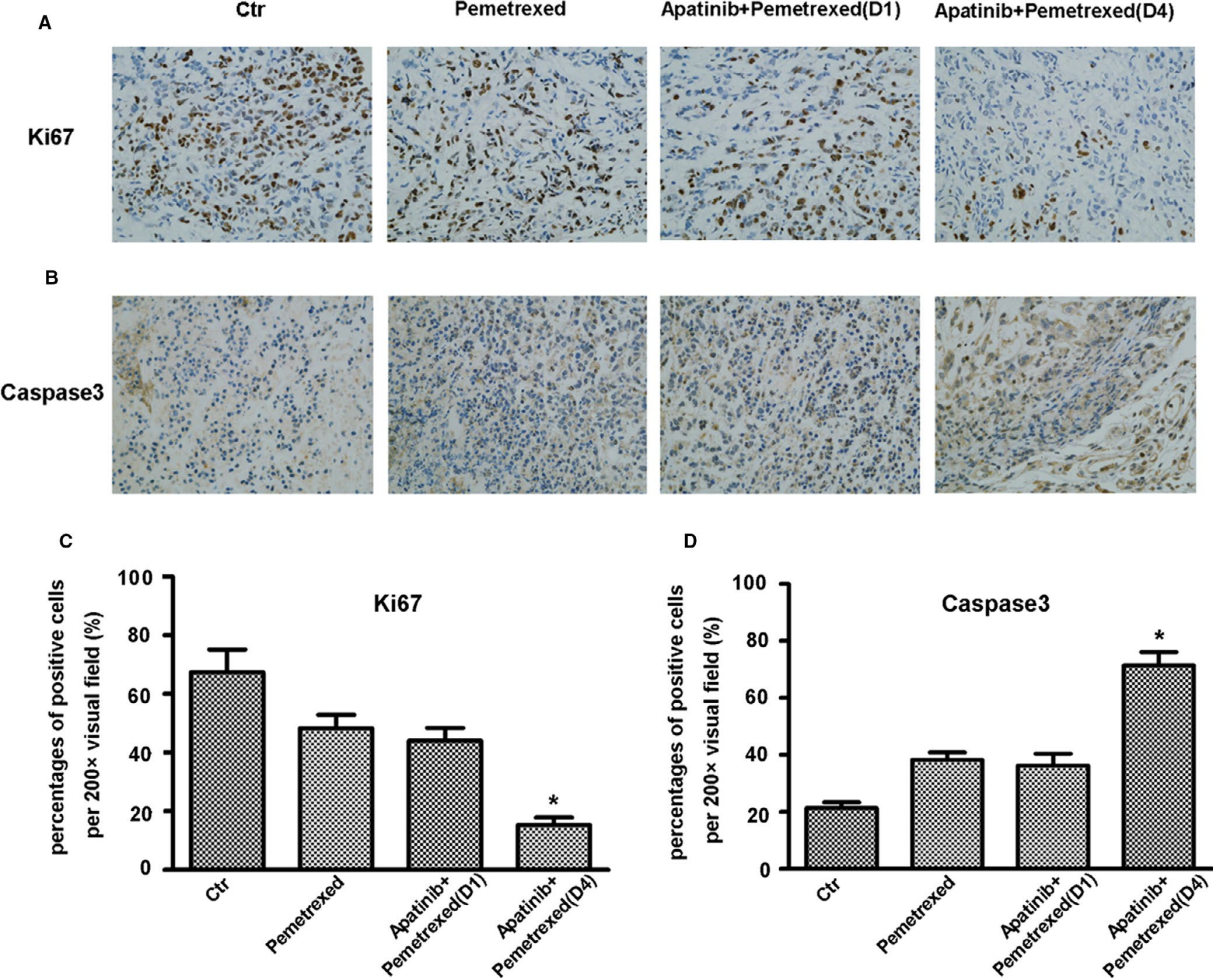
The immunohistochemical staining of the expression of Ki67 and c‐caspase3 in the different groups of xenografted‐tumor tissues (A and B) and the quantifications of the expression in each group (C and D)

**Figure 6:**
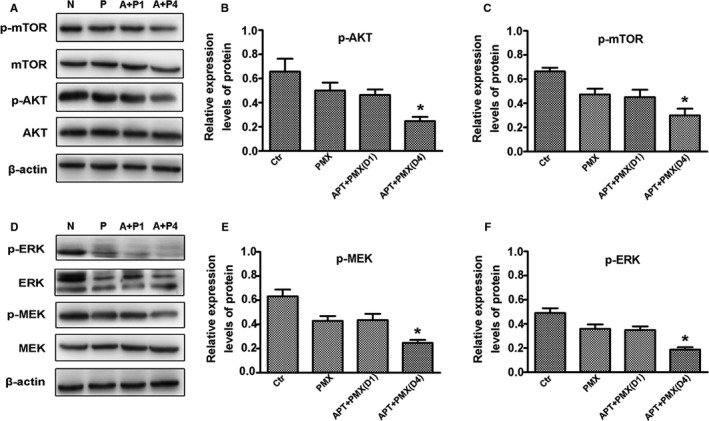
The signaling pathways in the different groups of xenografted‐tumor tissues. The expressions of p‐AKT and p‐mTOR were measured by Western blot (A, B, and C). The expressions of p‐MEK and p‐ERK were measured by Western blot (D, E, and F). Blots were representative of three independent experiments

In the four groups of transplanted tumor tissues, the expression of Ki67 in the transplanted tumor tissue of the sequential group was significantly reduced (Figure 5A,C), and the expression of caspase3 was significantly elevated, which was significantly different from those in the other three groups (*P* < .05) (Figure 5B,D). In the analysis of relevant signaling pathway factors, in the transplanted tumor tissue of the sequential group, the expression levels of p‐AKT and p‐mTOR were significantly lower than those in the other groups (Figure 6A) and there was a significant difference from those in the other three groups (Figure 6B,C). The expression levels of p‐MEK and p‐ERK were also significantly lower than those in the other groups (Figure 6D), there was significantly different from those in the other three groups (Figure 6E,F). The sequential therapy with low‐dose apatinib and pemetrexed may exert a biological effect through the above signaling pathways and inhibit the growth of xenografts.

### 3.7 The clinical efficacy and safety of apatinib sequential chemotherapy for patients with advanced non–small cell lung cancer

**Figure 7:**
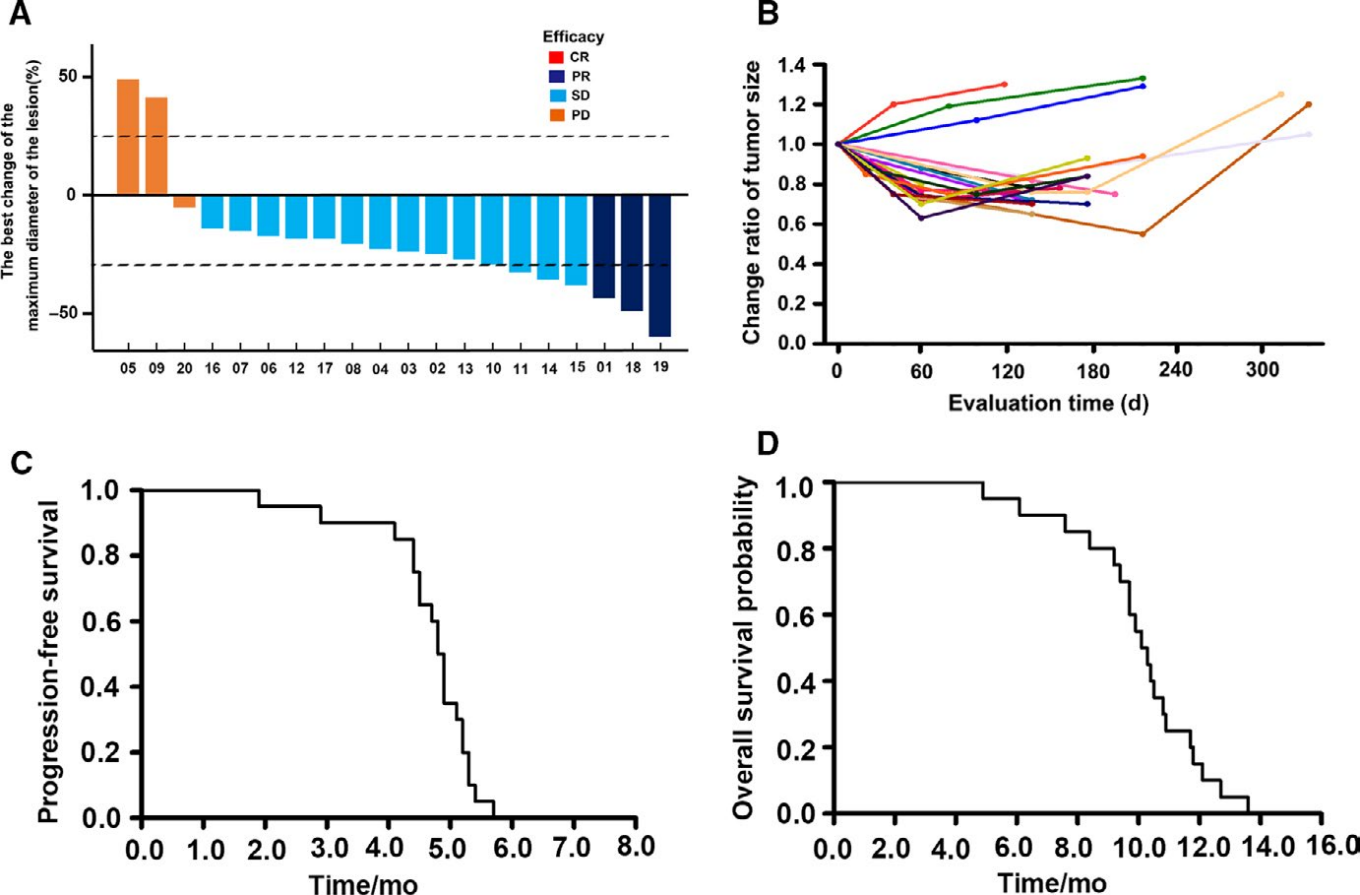
The progression‐free survival and overall survival probability of apatinib sequential chemotherapy for third‐line treatment in patients with advanced non‐small cell lung cancer

The general information of the patients is shown in Table 1. The responses of 20 patients with advanced non–small cell lung cancer treated with apatinib sequential chemotherapy were as follows: CR (n = 0), PR (n = 3), SD (n = 14), and PD (n = 3) (Figure 7A). The results of change ratio of tumor size were shown in Figure 7B. The ORR and DCR were 15.0% and 85.0%, respectively. The median PFS was 4.85 months, and the median OS was 10.2 months (Figure 7C,D). A toxicity assessment was performed to assess the toxicity of all patients; the grade 3/4 toxicity rate was 25%. No grade 4/4 toxicity was observed in any patients, and the common grade 3/4 adverse events were fatigue (n = 2), hypertension (n = 1), proteinuria (n = 1), nausea/vomiting (n = 1), and anemia (n = 1).

**Table 1:** The general information of patients

No.	Gender	Age years	Histology	Chemo therapy	Toxicity 3/4 grade	Efficacy	PFS (m)	OS (m)
1	Female	57	Adeno	DTX	No	PR	4.4	9.7
2	Male	62	Adeno	DTX	fatigue	SD	4.5	10.9
3	Male	58	Adeno	PEM	No	SD	4.9	10.8
4	Female	71	Squamous	DTX	No	SD	5.3	10.3
5	Female	66	Adeno	PTX	nausea	PD	1.9	4.9
6	Male	65	Squamous	PTX	anemia	SD	5.7	9.4
7	Female	62	Adeno	PEM	No	SD	4.8	10.4
8	Female	57	Adeno	PTX	No	SD	4.5	9.7
9	Female	63	Adeno	PEM	proteinuria	PD	2.9	7.6
10	Male	66	Squamous	GEM	hypertension	SD	5.2	10.5
11	Male	68	Adeno	DTX	No	SD	5.4	11.8
12	Female	70	Adeno	DTX	No	SD	4.4	9.2
13	Male	61	Adeno	PTX	No	SD	4.9	10.1
14	Male	50	Squamous	GEM	hepatic injure	SD	5.1	11.7
15	Female	69	Adeno	PEM	No	SD	5.3	13.6
16	Male	75	Adeno	PTX	No	SD	4.1	6.1
17	Female	68	Adeno	DTX	fatigue	SD	4.9	9.9
18	Male	49	Squamous	GEM	No	PR	5.2	12.1
19	Female	55	Adeno	DTX	No	PR	4.7	12.7
20	Male	67	Adeno	PEM	No	PD	4.8	8.4

Abbreviations: DTX, Docetaxel; GEM, Gemcitabine; OS, overall survival; PD, progressive disease; PEM, pemetrexed; PFS, progression‐free survival; PR, partial response; PTX, Paclitaxel; SD, stable disease.

## 4 DISCUSSION

Since the first antiangiogenic drug bevacizumab was used clinically, antiangiogenic therapy has become an important means of treating tumors.^32^ In particular, the application of small molecule oral antiangiogenic drugs has shown beneficial effects in tumor patients. Apatinib is a specific VEGFR‐2 receptor antagonist that competitively binds to the VEGFR‐2 receptor, blocks VEGF‐mediated signaling, inhibits tumor angiogenesis, and thereby controls tumor growth.^33^ At present, there are more than 30 clinical studies on the treatment of advanced lung cancer with apatinib. Most patients have advanced progressive non–small cell lung cancer, and a small number of patients have small cell lung cancer. Most of the treatments are focused on third‐line or higher treatment. Apatinib monotherapy also includes apatinib combined with chemotherapy or targeted drugs.^34^, ^35^, ^36^ In all clinical studies, monotherapy had poor efficacy. The median PFS was mostly shorter than 3 months, and the disease control rate was less than 50%.^37^ Therefore, apatinib monotherapy is no longer recommended to treat lung cancer. Based on the effect of apatinib combined with chemotherapy in the treatment of advanced lung cancer, combined treatment could improve the PFS and OS to a certain extent compared with those achieved with apatinib monotherapy, but the overall survival was still short.^30^ Strategies to increase the efficacy of apatinib are urgently needed.

**Figure 8:**
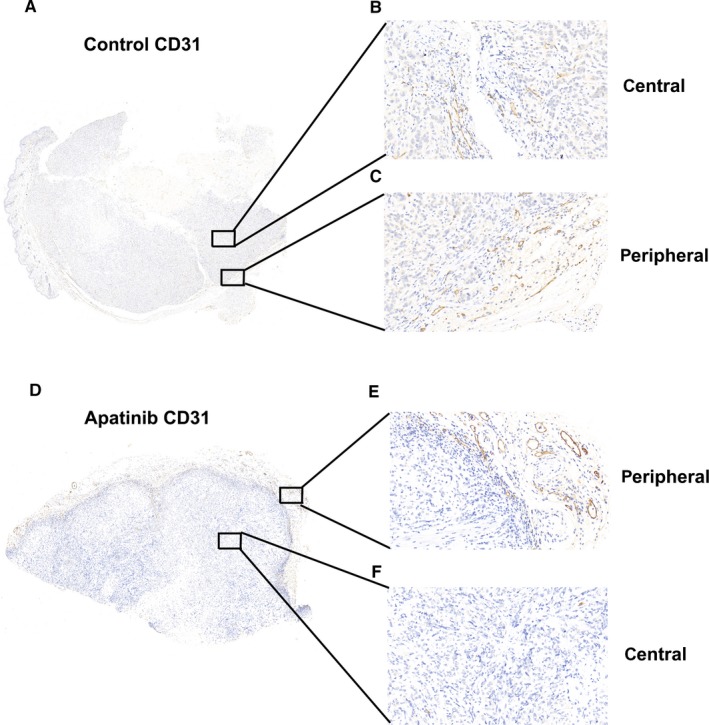
The effect of anti‐angiogenic drugs on the expression of CD31 in different parts of transplanted tumor tissue. (A and D) The overall expression of CD31 expression in transplanted tumor tissues of the control and apatinib groups. (B and C) The expression of CD31 in the central and peripheral part of transplanted tumor tissues of the control group. (E and F) The expression of CD31 in the peripheral and central part of transplanted tumor tissues of the apatinib group

The traditional view was that antiangiogenic drugs mainly inhibit the flow of tumor blood by inhibiting the formation of tumor blood vessels, thereby inhibiting the growth of tumors or "starving” the tumors. From the basic and clinical research points of view, this theory is correct. Antiangiogenic drugs can effectively control the growth of tumors for a period of time, but soon, tolerance occurs, tumors recur, and there is a so‐called "rebound phenomenon". How to avoid this phenomenon and improve the efficacy of antiangiogenic drugs is an urgent problem that needs to be solved in clinical practice.^38^, ^39^ Our previous study found that in a basic study of tumor‐bearing nude mice, the microvessel density of transplanted tumor tissue was significantly reduced within 2‐3 weeks after treatment with apatinib, indicating that apatinib can effectively control the growth of tumor blood vessels (Figure 8A,D). However, based on the immunohistochemistry results, there was "spatial heterogeneity" in the effect of apatinib on the microvessels of transplanted tumors. The angiogenic effect on the central tumor was strong (Figure 8B,F) and that in the peripheral tissues was weak (Figure 8C,E). The surrounding tissue became the source of tumor recurrence and rebound. Clinically, after the application of antiangiogenic drugs in lung cancer patients, some patients have voids, central necrosis in the tumor, and residual tumors in the periphery, further illustrating this situation. These findings also explain why antiangiogenic drug monotherapy has poor efficacy and relapse is common.

To address recurrences that develop shortly after antiangiogenic drugs monotherapy, combination therapy could be a possible strategy, including combined chemotherapy, targeted therapy, or immunotherapy. However, as mentioned above, the clinical results of apatinib combined with chemotherapy in the treatment of advanced lung cancer did not significantly improve the patients’ PFS and OS, which we believe may be related to the mode of administration. Conventional doses of antiangiogenic drugs can effectively reduce the formation of tumor angiogenesis. In combined treatment with chemotherapy drugs, due to the sharp reduction in tumor neovascularization, especially in the center of the tumor, the effective concentration of the local chemotherapy drugs was lower than intended, which strongly affected the sensitivity of the tumor to the chemotherapy drugs, resulting in poor treatment. Moreover, ischemia, hypoxia, and acidosis further affect the metabolism and transportation of chemotherapy drugs, resulting in poor therapeutic effects. Astrid A. M et al used positron emission tomography and radiolabelled docetaxel in patients with non–small cell lung cancer (NSCLC).^40^ In patients with NSCLC, bevacizumab reduced the flow rate and net flow rate of docetaxel within 5 hours, and these effects persisted after 4 days. These findings further illustrated that antiangiogenic drugs were prone to causing local chemotherapeutic drug transport and reducing the effective blood drug concentrations.

Based on these results, antiangiogenic drugs do not seem to be recommended for use in combination with chemotherapy drugs, and since 2005, when Jain first proposed the "vascular normalization theory", an increasing number of studies have confirmed this view. After treatment with antivascular drugs, the tumor blood vessels will appear "normalized" within a certain period of time, that is, the tumor blood vessels become more regular, the morphology of the pericytes is regular, and the basement membrane structure is intact, thereby improving the hypoxia and acidosis inside the tumor. This state reduces the interstitial pressure in the tumor tissue, thereby improving the transportation of the chemotherapeutic drug in the tumor, increasing the local effective concentration of the chemotherapeutic drug, and improving the curative effect of the chemotherapeutic drug. Our test results further confirmed this view. The tumor tissue releases a variety of factors to promote the growth of new blood vessels. VEGF is the most important angiogenic factor. Under the action of VEGF, tumor neovascularization grows rapidly, and vascular tortuosity, disorder, local expansion, leakages, and elevated interstitial tissue pressure were observed. Elevated pressure results in ischemia, hypoxia, and increased HIF expression, resulting in a series of biological effects. Apatinib is the most specific inhibitor of VEGFR‐2, which can effectively attenuate the effect of VEGF, thereby reducing the expression of HIF, regular cell morphology, and intact basement membrane structure. In our experiments, we found that after 3‐7 days of intervention, low‐dose apatinib significantly reduced the expression of HIF‐1α in the transplanted tumor tissue, the α‐SMA level in the pericytes was significantly elevated, and the vascular basement membrane was intact. Type IV collagen expression was significantly elevated. This finding also indicates that after low‐dose apatinib for 3‐7 days, the local hypoxia status in the transplanted tumor tissue was significantly improved, and the tumor vascular structure was relatively intact, thus achieving transient normalization. In the high‐dose apatinib group, we did not observe a clear window of vascular normalization. What is the reason for this? We used immunohistochemistry to detect the MVD in the transplanted tumor tissues. We found that in the high‐dose apatinib group, the MVD in transplanted tumor tissues was significantly reduced regardless of the length of intervention, especially in the center of the transplanted tumor. The reduced MVD and absence of new blood vessels indicated that there was no normalization. Therefore, we believe that the appearance of the normalization window of blood vessels is related not only to the timing of antiangiogenic drug treatment but also to the dose. High‐dose antiangiogenic drugs significantly inhibited tumor angiogenesis, leading to the failed normalization of blood vessels. These findings explain why in some studies, after an antivascular drug intervention, there was no "normalization of blood vessels", which in turn led to a reduction in the efficiency of chemotherapy drug transport and a reduction in the effective blood concentration in tumors. Therefore, only a suitable dose of antiangiogenic drugs may cause normalization of blood vessels within a certain period of time.

To further confirm the existence of vascular normalization, we used transmission electron microscopy to observe the ultrastructure of neovascularization in transplanted tumor tissue after apatinib intervention. The results further confirmed the normalization of vascularization at a low dose of apatinib for 3‐7 days. Under electron microscopy, we observed that after low‐dose apatinib treatment for 3 days and 7 days, the vascular structure of the transplanted tumor tissue was relatively complete and regular, more complete pericytes were observed peripherally, and the basement membrane structure of the blood vessels was relatively complete. In the control group and the high‐dose apatinib group, we found that the tumor blood vessels were disordered, the structure was incomplete, there were no mature pericytes, and the vascular basement membrane structure was incomplete.

Why does low‐dose apatinib intervention cause transient vascular normalization in the transplanted tumor vessels? We believe that as the local hypoxia improves, the expression of HIF is significantly reduced, and the change in HIF expression level is related to multiple cellular pathways that lead to changes in the levels of various factors. In particular, MMP‐2 and MMP‐9 are critical for the degradation of basement membrane type IV collagen, increased vascular leakage, and increased IFP.^41^ In the tumor tissue, the expression levels of MMP‐2 and MMP‐9 are elevated due to ischemia and hypoxia, and type IV collagen of the vascular basement membrane is degraded, resulting in destruction of the vascular structure. This study found that the expression of MMP‐2 and MMP‐9 in transplanted tumor tissues was significantly reduced during the 3‐7 days of low‐dose apatinib treatment, resulting in a significant reduction in the degradation of the vascular basement membrane and normalization of the tumor blood vessels in a short time.

To further confirm that combination chemotherapy in the time window of vascular normalization can effectively improve the therapeutic effect of chemotherapy drugs, we used a nude mouse xenograft model and the wild‐type lung cancer cell line A549 to construct a model. We found that the administration of pemetrexed chemotherapy on the 4th day after the low‐dose apatinib intervention significantly reduced the volume of the transplanted tumor; on the first day, the combination pemetrexed group experienced a much weaker antitumor effect than the first group. Pemetrexed was applied for 4 days. Immunohistochemistry and related cell signaling pathway expression studies were performed on transplanted tumors from different intervention groups and confirmed that low‐dose apatinib with sequential pemetrexed can effectively inhibit the proliferation of transplanted tumors and promote their apoptosis. Biological effects can be exerted through the PI3K‐AKT‐mTOR and MEK‐ERK‐MNK signaling pathways. This further indicates that after the application of a suitable dose of antiangiogenic drugs, the tumor vascular normalization time window does exist for a period of time, and the combination of chemotherapy drugs in this time window can effectively improve the efficacy of chemotherapy drugs.

In clinical practice, we selected 20 patients with advanced non–small cell lung and administered lower doses of apatinib combined with chemotherapy. The oral dose of apatinib was 250 mg for 5 days, and on the 6th day, chemotherapy drug treatment was administered every 21 days. The median PFS and OS were 4.75 months and 9.85 months, respectively. Compared with other third‐line treatments for advanced non–small cell lung cancer, this method led to a prolonged PFS and OS. However, due to the small sample size and lack of a similar control group, it is necessary to expand the sample size in the future, conduct randomized controlled studies, and further search for resistance. The best time to combine angiogenic drugs with chemotherapeutic drugs was determined.

In our clinical study, although we were not able to specifically monitor whether each patient actually achieved vascular normalization, our final data suggest that the oral administration of apatinib for a certain time and dosage can produce a short window of vascular normalization, and combination chemotherapy during this window can achieve better clinical treatment effects.

The dose and timing of apatinib for vascular normalization (250 mg/day for 5 days) were determined through animal data. However, the specific dose and timing of apatinib for patients to normalize their blood vessels must vary from person to person. Therefore, in future clinical research, DCE‐MRI and other examinations can be used to determine the window of vascular normalization. In addition, an individualized dosing strategy should be used, so that every patient can be treated in the accurate time window of vascular normalization.

## CONFLICT OF INTEREST

None declared.

## AUTHOR CONTRIBUTION

Mingtao Liu and Yu Li conceived and designed experiments; Mingtao Liu, Hui Li, Xiuxiu Wang, Lijun Jing, and Peng Jiang conducted the experiments; Mingtao Liu analyzed the data and wrote the manuscript; and Hui Li, Xiuxiu Wang, Lijun Jing, and Peng Jiang collected tissue samples and clinical data. All authors read and approved the final manuscript.

## Data Availability

The raw/processed data required to reproduce these findings cannot be shared at this time as the data also form part of an ongoing study.
